# Association among plasma 1,25(OH)_2_D, ratio of 1,25(OH)_2_D to 25(OH)D, and prostate cancer aggressiveness

**DOI:** 10.1002/pros.23824

**Published:** 2019-05-11

**Authors:** Swathi Ramakrishnan, Susan E. Steck, Lenore Arab, Hongmei Zhang, Jeannette T. Bensen, Elizabeth T. H. Fontham, Candace S. Johnson, James L. Mohler, Gary J. Smith, L. Joseph Su, Anna Woloszynska

**Affiliations:** ^1^ Department of Pharmacology and Therapeutics Roswell Park Comprehensive Cancer Center Buffalo New York; ^2^ Department of Epidemiology and Biostatistics Arnold School of Public Health, University of South Carolina Columbia South Carolina; ^3^ David Geffen School of Medicine University of California Los Angeles California; ^4^ Division of Epidemiology, Biostatistics, and Environmental Health University of Memphis Memphis Tennessee; ^5^ Department of Epidemiology Gillings School of Global Public Health, Lineberger Comprehensive Cancer Center, University of North Carolina at Chapel Hill Chapel Hill North Carolina; ^6^ School of Public Health Louisiana State University Health Sciences Center New Orleans Louisiana; ^7^ Department of Urology Roswell Park Comprehensive Cancer Center Buffalo New York; ^8^ Winthrop P. Rockefeller Cancer Institute, Department of Epidemiology, Fay W. Boozman College of Public Health University of Arkansas for Medical Sciences Little Rock Arkansas

**Keywords:** prostate cancer, racial disparities, vitamin D

## Abstract

**Background:**

African‐American (AA) men tend to present with more aggressive prostate cancer (Gleason score >7) than European‐American (EA) men. Vitamin D and its metabolites are implicated in prostate cancer biology with vitamin D deficiency, indicated by its metabolite levels in serum or plasma, usually observed in AA men.

**Objective:**

To determine if 1, 25‐dihydroxy vitamin D3 [1,25(OH)_2_D] plasma levels in AA and EA prostate cancer patients alter the risk of having aggressive prostate cancer.

**Design:**

Research subjects from the North Carolina‐Louisiana Prostate Cancer Project (AA n = 435 and EA n = 532) were included. Plasma metabolites 1,25(OH)_2_D and 25‐hydroxyvitamin D3 [25(OH)D] were measured using liquid chromatography with tandem mass spectrophotometry. Research subjects were classified into low (Gleason sum < 7, stage T1‐T2, and Prostate‐specific antigen (PSA) < 9 ng/mL) or high (Gleason sum > 8 or Gleason sum = 7 with 4 + 3, or PSA > 20 ng/mL, or Gleason sum = 7 and stage T3‐T4) aggressive disease.

**Results:**

Research subjects in the second and third tertiles of plasma levels of 1, 25(OH)_2_D had lower odds of high aggressive prostate cancer (AA [OR_T2vsT1_: 0.66, 95%CI: 0.39‐1.12; OR_T3vsT1_: 0.83, 95%CI: 0.49‐1.41] and EA [OR_T2vsT1_: 0.68, 95%CI: 0.41‐1.11; OR_T3vsT1_: 0.67, 95%CI: 0.40‐1.11]) compared with the first tertile, though confidence intervals included the null. Greater 1,25(OH)_2_D/25(OH)D molar ratios were associated with lower odds of high aggressive prostate cancer more evidently in AA (OR_Q4vsQ1_: 0.45, CI: 0.24‐0.82) than in EA (OR_Q4vsQ1_: 0.64, CI: 0.35‐1.17) research subjects.

**Conclusions:**

The 1,25(OH)_2_D/25(OH)D molar ratio was associated with decreased risk of high aggressive prostate cancer in AA men, and possibly in EA men. Further studies analyzing vitamin D polymorphisms, vitamin D binding protein levels, and prostatic levels of these metabolites may be useful. These studies may provide a better understanding of the vitamin D pathway and its biological role underlying health disparities in prostate cancer.

## 1 INTRODUCTION

Vitamin D is absorbed primarily through the skin and converted to its metabolites by a family of hydroxylase enzymes that include CYP27B1 and CYP24A1.^1^, ^2^ Vitamin D is converted to 25‐hydroxyvitamin D3 (25(OH)D) in the liver and to its active metabolite 1, 25‐dihydroxy vitamin D3 (1,25(OH)_2_D) in the kidney.^1^ 1,25(OH)_2_D binds the vitamin D receptor (VDR) that allows VDR to bind to specific DNA motifs of its target genes, such as p21 and p27.^3^, ^4^ Alterations in components of vitamin D metabolism, which include polymorphisms in the hydroxylase enzymes, can lead to varying serum vitamin D metabolite levels in individuals from different ethnic cohorts.^5^, ^6^, ^7^, ^8^ African‐American (AA) men and women usually present with low 25(OH)D levels compared with their European‐American (EA) counterparts,^9^, ^10^, ^11^ even though 1,25(OH)_2_D levels are similar by race.^12^ Skin pigmentation, decreased consumption of dairy products and fortified foods, lack of vitamin D supplementation, and elevated body mass index (BMI) have been related to low 25(OH)D levels in AAs.^11^, ^13^

AA men are more likely to be diagnosed with and/or die from aggressive prostate cancer than EA men.^14^ Differences in vitamin D metabolite levels have been suggested to play a role in prostate cancer disparities between AA and EA men.^15^, ^16^, ^17^, ^18^ 1,25(OH)_2_D negatively regulates CDK2^19^ or induces Rb1^20^ that result in cell cycle arrest in the G1 phase, to reduce prostate cancer cell proliferation in vitro. 1,25(OH)_2_D also induces cell cycle arrest and senescence in an IL‐1α‐dependent manner in prostate progenitor epithelial/stem cells isolated from adult mice in vitro.^21^ In vivo, dietary vitamin D or calcitriol supplementation reduced prostate cancer xenograft growth and increased 1,25(OH)_2_D serum levels.^22^ In another study, disruption of CYP24A1 activity increased 1,25(OH)_2_D levels and reduced prostate cancer growth in vitro and in vivo.^23^ However, the mechanism of action of 1,25(OH)_2_D was not defined well in these studies.

Most studies in prostate cancer measure vitamin D sufficient or deficient status in individuals by measuring 25(OH)D levels in the serum and not necessarily the active 1,25(OH)_2_D metabolite.^15^, ^16^, ^17^, ^18^ Our previous study showed that research subjects with the highest percentage of African ancestry had the lowest levels of 25(OH)D.^24^ While high 25(OH)D was associated with increased odds of high aggressive prostate cancer among AAs in PCaP, the relationship was modified by calcium intake and high 25(OH)D coupled with high calcium intakes decreased the odds of having high aggressive prostate cancer in AA men.^24^ In the current study, we investigated if the active metabolite 1,25(OH)_2_D levels and molar ratios of 1,25(OH)_2_D to 25(OH)D were associated with the risk of high aggressive prostate cancer in a racially diverse, case‐only study.

## 2 MATERIALS AND METHODS

### 2.1 Study population

A subset of research subjects from the North Carolina‐Louisiana Prostate Cancer Project (PCaP)^25^ was included in this study. A subset of n = 1000 research subjects who agreed to future use of their biological specimens were selected *a priori*. These subjects were selected before any measurements or analysis of 1,25(OH)_2_D or 25(OH)D. The institutional review boards at the University of North Carolina at Chapel Hill (UNC), and Louisiana State University Health Sciences Center and the Department of Defense Prostate Cancer Research Program approved the PCaP protocol. All research subjects consented to the release and use of their medical records and biologic samples. Residents of North Carolina (NC) and Louisiana (LA), between 40 to 79 years old, with a confirmed diagnosis of prostate adenocarcinoma and enrolled between 2004 and 2009 were included. The final study population consisted of 314 high aggressive and 683 low aggressive prostate cancer research subjects.

### 2.2 Data collection

A trained registered nurse conducted structured interviews, collected necessary specimens and measurements of research subjects at their home within 4 months of initial diagnosis, on average. Structured interviews were performed using a questionnaire that included a family history of prostate cancer, cancer screening history and vitamin supplementation among other variables listed in Table 1. The National Cancer Institute Diet History Questionnaire modified to accommodate Cajun, and Creole foods were used to inform about food portion sizes and intake frequency for a list of 124 food items. The Diet*Calc software was used to calculate the intake of nutrients and minerals including calcium and vitamin D.

**Table 1:** Descriptive statistics by aggressiveness and race

Characteristics	Low aggressive				High aggressive			
	AA (n = 260)		EA (n = 423)		AA (n = 175)		EA (n = 139)	
Continuous variables	Mean	SD	Mean	SD	Mean	SD	Mean	SD
Age, y	60.5	8.0	63.1	7.5	62.8	7.4	66.0	8.0
Body mass index, kg/m^2^	29.0	5.2	29.0	4.9	29.5	6.7	30.0	5.2
Total energy intake, kcal/d	2669.8	1354.5	2377.2	1168.8	3050.3	1589.2	2525.7	1293.6
Alcohol intake, g/d	17.3	45.8	15.0	44.9	28.8	92.4	18.4	52.6
Physical activity, MET‐hours/week	3.6	5.1	4.1	5.4	3.1	4.7	4.5	5.8
**Categorical variables**	**n**	**%**	**n**	**%**	**n**	**%**	**n**	**%**
Study site								
NC	128	49.2	212	50.1	84	48.0	64	46.0
LA (pre & post Katrina)	132	50.8	211	49.9	91	52.0	75	54.0
Family History of PrCa								
No affected 1st degree relative	171	71.9	286	72.4	118	74.7	107	83.0
One or more affected 1st degree relatives	67	28.2	109	27.6	40	25.3	22	17.0
Education								
Grad/professional degree	21	8.1	91	21.5	6	3.4	31	22.3
Some college or college graduate	82	31.5	173	40.9	50	28.6	59	42.5
High school grad or voc/tech school	90	34.6	127	30.0	54	30.9	32	23.0
Less than than 8th grade or some high school	67	25.8	32	7.6	65	37.1	17	12.2
Screening History								
0 screenings	83	31.9	63	14.9	91	52.0	23	16.6
0–7 screenings	119	45.8	189	44.7	50	28.6	66	47.5
More than 7 screenings	58	22.3	171	40.4	34	19.4	50	36.0
Smoking Status								
Nonsmoker	100	38.5	165	39.0	39	22.3	54	38.9
Former smoker	115	44.2	219	51.8	94	53.7	73	52.5
Current smoker	45	17.3	39	9.2	42	24.0	12	8.6
NSAID use								
No	121	46.5	131	31.0	67	38.5	43	31.2
Yes	139	53.5	292	69.0	107	61.5	95	68.8
Season								
Winter (21 Dec‐22 Mar)	47	18.1	80	18.9	20	11.4	22	15.8
Spring (21 Mar‐20 Jun)	50	19.2	81	19.2	36	20.6	28	20.1
Fall (21 Sep‐20 Dec)	117	45.0	201	47.5	91	52.0	66	47.5
Summer (21 Jun‐20 Sep)	46	17.7	61	14.4	28	16	23	16.6
African ancestry								
High African ancestry (>0.95)	163	63.2	0	0	113	66.5	0	0
Medium African ancestry (0.85 ‐ <0.95)	39	15.1	0	0	25	14.7	0	0
Low African ancestry (<0.85)	56	21.7	0	0	32	18.8	0	0
White	0		408	100.0	0		137	100
Marital Status								
Single/separated/divorced/widowed	82	31.5	54	12.8	61	34.9	31	22.3
Married/living with partner	178	68.5	369	87.2	114	65.1	108	77.7
PrCa treatment in preceding 90 d								
No	105	45.5	171	43.0	53	33.1	31	23.9
Yes	126	54.6	227	57.0	107	66.9	99	76.2

Abbreviations: AA, African‐American; EA, European‐American; LA, Louisiana; NC, North Carolina; NSAID, Nonsteroidal Anti‐inflammatory Drugs; PrCa, prostate cancer.

### 2.3 Vitamin D measurements

Fasting venous blood was drawn in foil‐wrapped EDTA tubes and transported at 4°C to the Blood and Tissue Procurement Core laboratory at LSU or the BioSpecimen Processing Facility at UNC that processed the samples into serum, plasma, and DNA on the same or next day. These samples were aliquoted and stored at −80°C. 25(OH)D and 1,25(OH)_2_D are stable for up to 10 years when stored at −20°C.^26^ In this study, plasma samples were stored for up to 8 years before any 1,25(OH)_2_D or 25(OH)D analysis. Heartland Assays, Inc used liquid chromatography with tandem mass spectrophotometry to measure plasma levels of 1,25(OH)_2_D and 25(OH)D.

### 2.4 Outcome assessment

In PCaP, research subjects were categorized into the following three categories of aggressiveness^25^: (1) High aggressive cases: Gleason sum ≥ 8, or PSA > 20 ng/mL at diagnosis, or Gleason sum = 7 and stage T3‐T4; (2) low aggressive cases: Gleason sum < 7 and diagnosed at stage T1‐T2 and PSA < 10 ng/ml at diagnosis; and (3) intermediate aggressive cases: all other cases. For the current study, all PCaP research subjects diagnosed with high aggressive cancer and those intermediate aggressive cancer research subjects who had Gleason sum = 7 with primary Gleason pattern 4 were combined and referred to as the high aggressive group. A random sample of low aggressive cases with Gleason sum > 7, stage T1‐T2, and PSA < 9 ng/mL were selected from the overall PCaP study population for comparison to the high aggressive group at an approximate 2:1 ratio of low aggressive to high aggressive research subjects.

### 2.5 Statistical analysis

Means and standard deviations were calculated for continuous variables. Frequencies and percentages were calculated for categorical variables. Crude and multivariable logistic regression analyses were used to examine associations between 1,25(OH)_2_D levels in AA and EA research subjects with high aggressive prostate cancer, with low aggressive cases serving as the comparison group. Race‐specific cut‐offs for 1,25(OH)_2_D were set to group research subjects into tertiles due to different plasma distributions between AA and EA research subjects, with the lowest tertile serving as the referent. To replicate our previous analyses in a larger sample of PcaP research subjects, we re‐examined the association between tertiles of 25(OH)D and high aggressive prostate cancer by race in this smaller subset. Molar ratios of plasma 1,25(OH)_2_D to 25(OH)D were calculated and divided into race‐specific quartiles. The regression models were adjusted for parameters described at the end of Tables 2, 3, 4, 5 that includes age, BMI, education, PSA screening history, smoking, alcohol intake, nonsteroidal anti‐inflammatory drug (NSAID) use, study site, the season of blood draw, physical activity and total energy intake. Joint effects of 1,25(OH)_2_D and 25(OH)D were examined using the lowest category of each as the referent group and comparing all other joint categories using multivariate logistic regression. All the analysis were evaluated at *p* < 0.05 (two‐tailed) using SAS version 9.3 (SAS Institute, Cary, NC).

**Table 2:** Association between plasma 1,25(OH)_2_D and prostate cancer aggressiveness by race

Plasma 1,25(OH)_2_D pg/mL, tertiles	n, High/low aggressive	Crude OR^1^	95%CI^1^	Adjusted OR^2^	95%CI^2^
African‐Americans					
<23.98	69/85	1.00	Ref.	1.00	Ref.
23.98 – <31.18	50/86	0.73	0.45‐1.18	0.66	0.39‐1.12
≥31.18	56/89	0.88	0.55‐1.42	0.83	0.49‐1.41
European‐Americans					
<25.40	61/141	1.00	Ref.	1.00	Ref.
25.40 ‐ <32.50	40/140	0.67	0.42‐1.08	0.68	0.41‐1.11
≥32.50	38/142	0.65	0.40‐1.06	0.67	0.40‐1.11

Abbreviations: CI, confidence interval; OR, odds ratio.

^1^ Adjusted for age.

^2^ Adjusted for age, education, alcohol intake, smoking status, season of blood draw, PSA screening history, physical activity, energy intake, NSAIDs use, study site and BMI.

**Table 3:** Association between plasma 25(OH)D and prostate cancer aggressiveness by race in 1000 PCaP research subjects who have data available on 1,25(OH)_2_D

Plasma 25(OH)D ng/mL, tertiles	n, High/low aggressive	Adjusted OR^1^	95%CI^1^
African‐Americans			
<13.30	52/91	1.00	Ref.
13.30 ‐ <18.90	57/90	1.54	0.90‐2.64
≥18.90	66/79	1.60	0.93‐2.75
European‐Americans			
<21.14	52/146	1.00	Ref.
21.14 ‐ <26.67	48/147	0.95	0.59‐1.54
≥26.67	39/130	0.86	0.51‐1.45

Abbreviations: CI, confidence interval; OR, odds ratio; PCaP, prostate cancer project.

^1^ Adjusted for age, education, alcohol intake, smoking status, season of blood draw, PSA screening history, physical activity, energy intake, NSAIDs use, study site and BMI.

**Table 4:** Association between molar ratio of plasma 1,25(OH)_2_D to 25(OH)D and prostate cancer aggressiveness by race

1,25(OH)_2_D to 25(OH)D molar ratio, quartiles	n, High/low aggressive	Crude OR^1^	95%CI^1^	Adjusted OR^2^	95%CI^2^
African‐Americans					
<1.29	69/64	1.00	Ref.	1.00	Ref.
1.29 ‐ <1.74	39/64	0.54	0.32‐0.91	0.52	0.29‐0.93
1.74 ‐ <2.19	27/64	0.43	0.24‐0.76	0.40	0.22‐0.74
≥2.19	35/65	0.56	0.32‐0.98	0.45	0.24‐0.82
European‐Americans					
<0.97	41/106	1.00	Ref.	1.00	Ref.
0.97 ‐ <1.20	38/105	0.99	0.58‐1.69	1.08	0.62‐1.87
1.20 ‐ <1.50	33/105	0.89	0.51‐1.54	0.93	0.53‐1.63
≥1.50	25/106	0.66	0.37‐1.19	0.64	0.35‐1.17

Abbreviations: CI, confidence interval; OR, odds ratio.

^1^ Adjusted for age.

^2^ Adjusted for age, education, alcohol intake, smoking status, season of blood draw, PSA screening history, physical activity, energy intake, NSAIDs use, study site and BMI.

**Table 5:** Adjusted^a^ odds ratios for joint tertiles of plasma 25(OH)D and 1,25(OH)_2_D by race

25(OH)D tertiles^b^	1,25(OH)_2_D tertiles^c^					
	1		2		3	
	High/low aggressiveness	OR (95%CI)	High/low aggressiveness	OR (95%CI)	High/low aggressiveness	OR (95%CI)
African‐Americans						
1	26/43	1.00 Ref.	17/29	1.07 (0.46‐2.50)	9/19	0.86 (0.30‐2.48)
2	25/31	2.09 (0.94‐4.64)	12/28	0.73 (0.29‐1.89)	20/31	1.84 (0.80‐4.25)
3	18/11	3.03 (1.12‐8.22)	21/29	1.47 (0.63‐3.42)	27/39	1.24 (0.56‐2.73)
European‐Americans						
1	33/72	1.00 Ref.	12/49	0.64 (0.29‐1.39)	7/25	0.81 (0.30‐2.16)
2	18/40	1.16 (0.56‐2.40)	17/56	0.71 (0.34‐1.46)	13/51	0.64 (0.29‐1.39)
3	10/29	0.78 (0.32‐1.89)	11/35	0.73 (0.31‐1.71)	18/66	0.69 (0.33‐1.43)

Abbreviations: CI, confidence interval; OR, odds ratio.

^a^ Adjusted for age, education, alcohol intake, smoking status, season of blood draw, PSA screening history, physical activity, energy intake, NSAIDs use, study site and BMI.

^b^ Tertile cutpoints: African‐Americans: T1 < 13.30; 13.30 ≤ T2 < 18.90; T3 ≥ 18.90 ng/mL; European‐Americans: T1 < 21.14; 21.14 ≤ T2 < 26.67; T3 ≥ 26.67 ng/mL.

^c^ Tertile cutpoints African‐Americans: T1 < 23.98; 23.98 ≤ T2 < 31.18; T3 ≥ 31.18; European‐Americans: T1 < 25.40; 25.40 ≤ T2 < 32.50; T3 > 32.50.

## 3 RESULTS

### 3.1 Demographics of research subjects

AA and EA research subjects in both low and high aggressive prostate cancer had similar representation from NC and LA (46%‐50%). Fall interviews (>40%) were more common than the other three seasons. Less than 20% of AA research subjects had <0.85 African ancestries as determined by ancestry informative markers. At least 60% of EA and AA research subjects had high school‐level education, but less than 10% of AA and 25% of EA research subjects had a graduate or professional degree. A higher percentage of EA research subjects were married or living with a partner compared with AA research subjects (19% and 12% difference in low and high aggressive categories, respectively). A greater percent of AA research subjects were current smokers compared with EA research subjects in both aggressiveness categories. In low aggressive prostate cancer, AA and EA research subjects had a similar family history of prostate cancer. However, in high aggressive prostate cancer, a higher proportion of AA research subjects had a family history of prostate cancer compared with EA research subjects. More AA research subjects had not been screened than EA research subjects in the high aggressive category.

### 3.2 Increased plasma 1,25(OH)_2_D levels associated with reduced odds of high aggressive prostate cancer in AA and EA men

**Figure 1:**
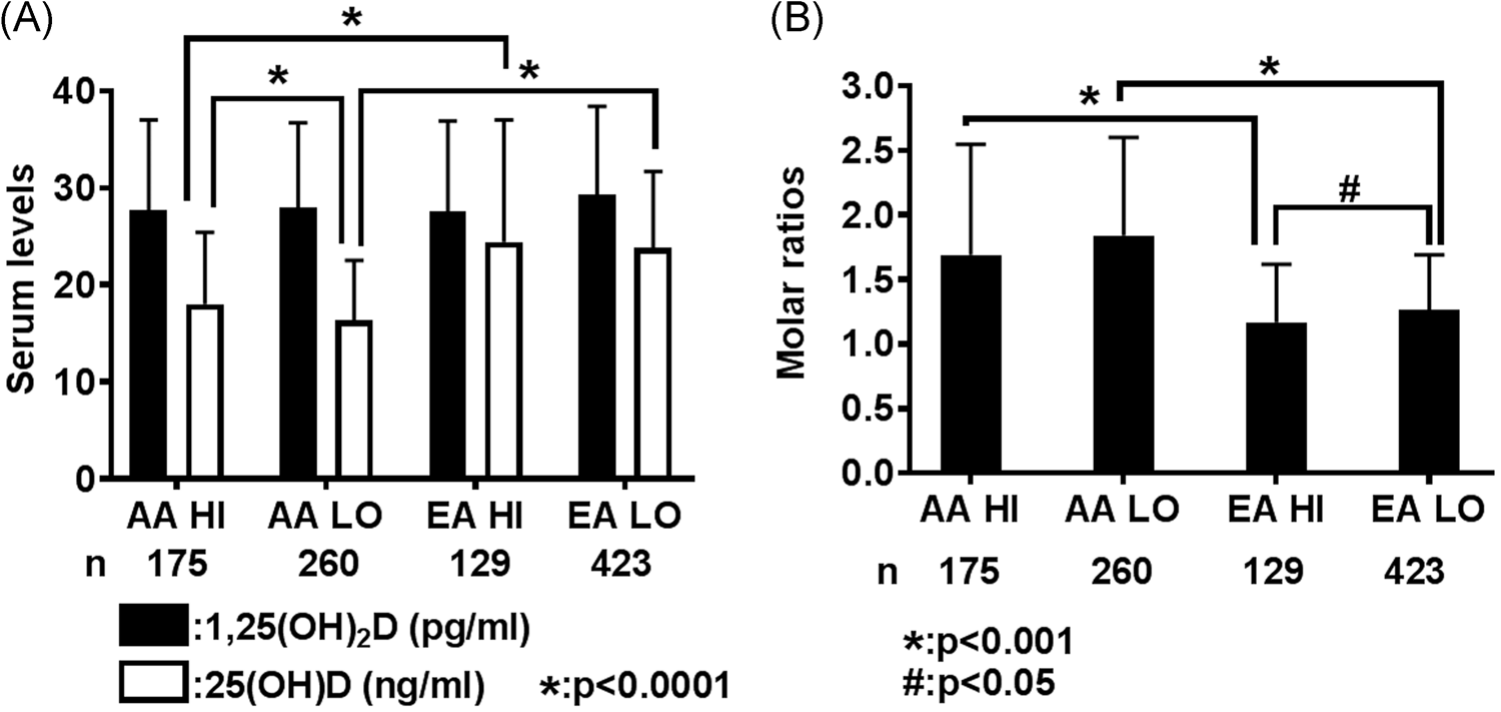
Plasma levels and molar ratios of 1,25(OH)_2_D and 25(OH)D. A, Mean ± standard deviations of plasma 1,25(OH)_2_D (pg/mL) and 25(OH)D (ng/mL) by race and aggressiveness. 25(OH)D levels in AA research subjects are lower compared with EA research subjects. B, Mean ± standard deviations of 1,25(OH)_2_D/25(OH)D molar ratio by race and aggressiveness. The molar ratios in AA research subjects are significantly higher compared with EA research subjects. AA, African‐American; EA, European‐American

Mean plasma levels of 1,25(OH)_2_D were similar between low aggressive AA (28.0 ± 8.7 pg/mL) and EA (29.3 ± 9.1 pg/mL) research subjects and high aggressive AA (27.7 ± 9.3 pg/mL) and EA (27.6 ± 9.3 pg/mL) research subjects (Figure 1A). Multivariable‐adjusted odds ratios in both AA and EA research subjects indicated that higher 1,25(OH)_2_D levels in the plasma were associated with reduced odds of high aggressive prostate cancer, though confidence intervals included the null (Table 2), and there was no evidence of a dose‐response association.

### 3.3 Odds of high aggressive prostate cancer were lower with increased 1,25(OH)_2_D/25(OH)D molar ratio

First, we reanalyzed the 25(OH)D plasma levels and their relation to prostate cancer aggressiveness in the subset of research subjects^24^ included in this study. We find similarly increased odds of high aggressive prostate cancer among AA research subjects for higher compared with lower 25(OH)D, although the results were not statistically significant probably given the reduced sample size (Table 3). Low aggressive AA (1.84 ± 0.76) and EA (1.26 ± 0.43) research subjects had higher molar ratios compared with high aggressive AA (1.69 ± 0.86) and EA (1.17 ± 0.45) research subjects (Figure 1B). AA research subjects, on average, had higher 1,25(OH)_2_D molar ratios compared with EA research subjects. This difference could be attributed to lower 25(OH)D plasma levels in AA compared with EA men (Figure 1A). In AA research subjects, both crude and adjusted models showed reduced odds of high aggressive prostate cancer with increasing molar ratios of 1,25(OH)_2_D/25(OH)D (Table 4). In EA research subjects, crude and adjusted OR did not show dramatic reductions in high aggressive prostate cancer with increasing molar ratio (Table 4). Only the fourth quartile of molar ratio (greater than 1.50) among EAs showed decreased odds of high aggressive prostate cancer (adjusted OR_Q4vsQ1_: 0.66, CI: 0.35‐1.17). Additionally, we examined the joint results of 1,25(OH)_2_D and 25(OH)D by race and found that the highest odds of high aggressive prostate cancer is among men in the joint category of low 1,25(OH)_2_D+high 25(OH)D [OR, 3.03 (95%CI, 1.12‐8.22) as compared with the referent group of low 1,25(OH)_2_D+low 25(OH)D] (Table 5).

## 4 DISCUSSION

Our study shows that increased plasma 1,25(OH)_2_D/25(OH)D molar ratios, and possibly 1,25(OH)_2_D levels, are associated with decreased odds of high aggressive prostate cancer. The association tended to be stronger, and the molar ratio values were higher among AA than EA research subjects, likely due to lower 25(OH)D levels among AA research subjects. Quartile cutpoints were set based on low aggressive prostate cancer research subjects within each race and results suggest one would need at least greater than or equal to 1.50 molar ratio to see a reduced association with prostate cancer aggressiveness.

Experimental studies have reported that 1,25(OH)_2_D is involved in prostate tumorigenesis. 1,25(OH)_2_D in prostate epithelium positively correlated with microRNAs 100 and 125b that showed tumor suppressive properties in vitro.^27^ The same group showed that 1,25(OH)_2_D can regulate microRNAs and DICER protein in prostatic stroma that positively associates with biochemical recurrence.^28^ These data suggest that 1,25(OH)_2_D can have diverse effects on both prostatic epithelial and stromal cells and this cross‐talk may be significant in prostate cancer biology. Prostate cancer organoids derived from AA patients will be a useful tool to study the effects of 1,25(OH)_2_D on prostate cancer cells in the context of a microenvironment.^29^

Clinically, there are conflicting reports about the use of vitamin D in prostate cancer, most of which are focused on EA prostate cancer patients. For example, a randomized trial examining vitamin D intake before radical prostatectomy in men with low Gleason score (6 or 7) showed that supplementation positively correlated with increased 25(OH)D and 1,25(OH)_2_D in both serum and prostate.^30^ Increased 25(OH)D and 1,25(OH)_2_D levels in the prostate negatively correlated with a proliferation marker, Ki‐67 and parathyroid hormone.^30^ However, a phase III ASCENT trial using a combination of docetaxel and calcitriol for castrate‐resistant prostate cancer patients was terminated due to reduced survival compared with the placebo arm.^31^ Earlier trials that used docetaxel once every 3 weeks showed superior survival rates compared with weekly docetaxel.^31^ Therefore, the authors suggested that weekly docetaxel schedule and not calcitriol was the possible reason for reduced survival in the treatment arm compared with the placebo arm.^31^

Trials that include AA men have provided contradictory evidence about the role of vitamin D in prostate cancer etiology. A study of healthy AA men (between 40‐60 years of age) supplemented with 1000, 2000, or 4000 IU/d of vitamin D and 200 mg calcium for 3 months showed no differences in PSA levels.^32^ However, longer‐term follow‐up studies are needed to examine the effects on prostate cancer. Results from the recently published VITAL study show that vitamin D supplementation has no role in preventing new cancers.^33^, ^34^ However, studies involving cancer patients and the impact of vitamin D supplementation on cancer progression in such patients is largely unknown. Vitamin D supplementation at 4000IU per day showed a reduction in the number of positive cores compared with matched historical controls in a small patient cohort that included EA and AA men with low‐risk disease.^35^ Another group reported that serum 1,25(OH)_2_D levels did not differ between AA and EA prostate cancer patients with localized disease, but 1,25(OH)_2_D levels were higher in prostates of AA compared with EA patients.^12^ The authors attributed this difference to a megalin mediated endocytosis process by which vitamin D metabolites are absorbed in a race‐ and prostate‐specific manner.^12^ The above‐mentioned studies in EA and AA men were performed at different stages of prostate cancer in patients with different treatment modalities, potentially explaining the different results found in various trials. Additional research is needed at the prostatic level to obtain a better understanding of vitamin D and its metabolites in AA and EA men.

Activities of plasma metabolites may be affected by other alterations in the vitamin D pathway. Vitamin D binding protein sequesters free circulating 1,25(OH)_2_D that is required to activate VDR. A recent study suggested that the risk of aggressive prostate cancer in AA men inversely correlated with vitamin D binding protein rather than 25(OH)D levels.^18^ Therefore, investigating the levels of vitamin D binding protein in the context of supplementation and serum/prostate metabolite levels may provide a more accurate picture of this pathway in prostate cancer and its role in racial disparities.^18^ VDR polymorphisms and prostate cancer aggressiveness in AA men are linked^16^, ^36^ with the risk of vitamin D deficiency.^37^ 1,25(OH)_2_D absorption may increase due to increased parathyroid hormone (PTH) 38 that is related to low circulating calcium levels.^39^ Increased 1,25(OH)_2_D levels can negatively regulate PTH, which can cause further activation or absorption of 25(OH)D.^38^ In both EA and AA populations, 25(OH)D and 1,25(OH)_2_D levels negatively correlate to PTH.^40^ This signaling feedback due to calcium and PTH adds another layer of complexity to vitamin D absorption, metabolism and downstream signaling.

Our study is strengthened by the use of rapid case ascertainment to identify a population‐based sample of approximately equal numbers of AA and EA men with newly‐diagnosed prostate cancer. Limitations include the use of only one measurement of plasma vitamin D metabolites that was collected after diagnosis, so we cannot rule out the possibility that disease status may have affected vitamin D metabolite concentrations. As in any observational study, residual or unmeasured confounding are possible limitations.

## 5 CONCLUSIONS

Our study showed that higher levels of the 1,25(OH)_2_D/25(OH)D molar ratio, and possibly of the active 1,25(OH)_2_D metabolite, were inversely related to odds of high aggressive prostate cancer, particularly in AA men. But the complex nature of the vitamin D pathway, its cross‐talk with stromal components and its dependence on other nutritional elements warrant careful analysis in relation to health disparities that occur in prostate cancer.

## AUTHOR CONTRIBUTIONS

AW, SES, LA, and CSJ conceived and designed experiments. AW, SES, LA, CSJ, HZ, and JTB performed experiments. AW, SES, LA, and HZ analyzed the data. AW, SES, LA, CSJ, HZ, JTB, ETHF, JLM, GJS, and LJS contributed reagents/materials/analysis tools. SR, AW, SES, LA, HZ, JTB, ETHF, JLM, CSJ, and LJS wrote the paper.
